# Pattern-Reversal Visual Evoked Potentials Tests in Persons with Type 2 Diabetes Mellitus with and without Diabetic Retinopathy

**DOI:** 10.1155/2020/1014857

**Published:** 2020-08-24

**Authors:** Raghda S. Al-Najjar, Nehaya M. Al-Aubody, Salah Z. Al-Asadi, Majid Alabbood

**Affiliations:** ^1^Department of Physiology, Al-Zahraa College of Medicine, University of Basra, Basra 61004, Iraq; ^2^Department of Ophthalmology, Basra College of Medicine, University of Basra, Basra 61004, Iraq; ^3^Department of Medicine, Al-Zahraa College of Medicine, University of Basra, Basra 61004, Iraq

## Abstract

**Background:**

Currently, diabetic retinopathy (DR) has a wide recognition as a neurovascular rather than a microvascular diabetic complication with an increasing need for enhanced detection approaches. Pattern-reversal visual evoked potentials (PRVEPs) test, as an objective electrophysiological measure of the optic nerve and retinal function, can be of great value in the detection of diabetic retinal changes.

**Objectives:**

The use of two sizes of checkerboard PRVEPs testing to detect any neurological changes in persons with type 2 diabetes mellitus (T2DM) with and without a clinically detected DR. Also, to compare the results according to the candidate age, duration, and glycemic status of T2DM.

**Methods:**

This study included 50 candidates as group A with T2DM and did not have a clinically detected DR and 50 candidates as group B with T2DM and had a clinically detected early DR and 50 candidates as controls who were neither diabetic nor had any other medical or ophthalmic condition that might affect PRVEPs test results. The PRVEPs were recorded in the consultant unit of ophthalmology in Almawani Teaching Hospital. Monocular PRVEPs testing of both eyes was done by using large (60 min) and small (15 min) checks to measure N75 latency and P100 latency and amplitude.

**Results:**

There was a statistically significant P100 latency delay and P100 amplitude reduction in both groups A and B in comparison with the controls. The difference between groups A and B was also significant. In both test results of groups A and B, the proportions of abnormal P100 latency were higher than those of P100 amplitude with a higher abnormal proportions in 15 min test.

**Conclusions:**

The PRVEP test detected neurological changes, mainly as conductive alterations affecting mostly the foveal region prior to any overt DR clinical changes, and these alterations were heightened by the presence of DR clinical changes.

## 1. Background

In the recent past, diabetic retinopathy (DR) is frequently categorized as a microvascular complication of diabetes mellitus (DM). However, in the last few years, DR is recognized as a neurovascular impairment or sensory neuropathy subsequent to the neurovascular impairment [1]. It is well documented that hyperglycemia and its related metabolic abnormalities have a major harmful effect on retinal neurovascular unit including neuronal, vascular, glial, and immune cells, and not just a microvascular effect. This hypothesis opens a new window to manage DR [2]. Many studies showed that electrophysiological procedures are sensitive tools in the early identification of diabetic neural alterations way before the clinical vascular changes become apparent on fundoscopy. Albeit, its use in regular screening is still low and have obtained a much less attention than the tests for diabetic peripheral neuropathy [3, 4].

The visual evoked potentials (VEPs) test is the primary tool and is superior to the magnetic resonance imaging (MRI) in assessing the functional integrity of the anterior visual pathways [5]. The pattern-reversal VEPs (PRVEPs) test is the standard and ideal modality for most clinical uses as it is less variable in timing and waveform than other VEP modalities. The use of large and small size checks is recommended by the International Society for Clinical Electrophysiology of Vision (ISCEV) standards [6]. The large size (60 min) mainly stimulates the retinal neural elements responsible for peripheral vision (parafovea), while the small size (15 min) mainly stimulates the retinal neural elements responsible for central vision (fovea) [7, 8].

The most prominent component of PRVEPs wave is the P100 as a positive peak with relatively minimal variability. The increased P100 latency is an indicator for a retinocortical conduction decrement as occurring in the demyelinating process. On the other hand, the P100 amplitude and waveform abnormalities may indicate axon loss in the visual pathway [9].

The aim of this study was to detect any neurological changes in persons with type 2 diabetes mellitus (T2DM) with and without a clinically detected DR through the use of two sizes of checkerboard PRVEP testing and also, to compare the results according to the candidates age, duration, and glycemic status of T2DM.

## 2. Subjects and Methods

### 2.1. Study Design

This is a prospective study conducted in Basra Governorate, Iraq, from December 1, 2017, to October 1, 2018. All candidates were interviewed, and informed consents were taken from them. The study included 150 participants randomly who attended the ophthalmology consultant unit in Almawani Teaching Hospital. The age of the candidates was restricted to forty years and above at time of DM first diagnosed to limit the study to T2DM [10]. The candidates were divided into group A which included 50 persons with T2DM and did not have a clinically detected DR and group B which included 50 persons with T2DM and had a clinically detected mild-moderate nonproliferative DR (NPDR) [11] (Supplementary Figure 1) and 50 candidates as the control group who were free from DM and did not have any of the exclusion criteria. Both eyes of the controls and group A were included, while only the eyes which had the clinical features of mild-moderate NPDR [11] were included in group B.

The PRVEPs were recorded using the RETI-port/scan 21 machine (Roland Consult, Brandenburg/Havel, Germany). It was done according to ISCEV standards [6], by using a full field pattern of black and white checks with central red fixation point. The checkerboard stimulus was of two sizes, large (60 min) and small (15 min) size checks. Monocular recording of both eyes were done by using a single-channel electrode of gold-plated type, with a four-channel amplifier whose band-pass filters were set at 1–50 Hz. The contrast was 97%, the plot time was (300msec), and the stimulus frequency was 1.53872 reversals per second. These test parameters were customized by the manufacturer and designated to measure the N75 latency, P100 latency, and amplitude. In this study, we will concentrate on P100 components as P100 is a prominent feature with relatively minimal variability [6].

### 2.2. Exclusion Criteria

Significant ocular diseases such as severe NPDR, proliferative DR, macular disease, vitreous opacities, visually significant cataract, glaucoma, optic neuropathy disease, best-corrected visual acuity less than 20/20, and amblyopia, all these conditions were excluded from the study. Any medical illness that can affect PRVEPs findings such as multiple sclerosis, epilepsy, thyroid disease, type 1 DM (T1DM), patients with a past history of head trauma or cerebrovascular accident, and uncontrolled hypertension (blood pressure (BP) above 140/90 mmHg) were excluded. In addition, alcoholics and drug addicts using such as heroin, morphine, cough syrups, pain killers, and sedatives (due to their negative impact on neural transmission) [12] and pregnant women were also excluded.

### 2.3. Data Collection

Each candidate underwent a thorough history taking, BP, weight, height, fasting plasma glucose (FPG), and glycated hemoglobin (HbA1c) measurement, and a comprehensive ophthalmic examination including refraction and visual acuity, intraocular pressure (IOP), and anterior and fundus segment examinations after mydriasis.

### 2.4. Subjects and Testing Room Preparation

Verbal consents were taken from all subjects who had a briefing about the procedure and instructed to fast the night before the tests and to avoid hair oils and cycloplegic drops. Subjects were seated comfortably in a stable position approximately 100 cm away from the monitoring screen, and the tested eye was in a proper alignment to the central fixation point with a precise focusing on it during testing. Subjects with refractive errors were asked to wear their corrective glasses. The testing room was maintained quiet and dim lighted with no other operating instruments during the test.

### 2.5. Electrode Placement

The study was done according to the International 10/20 system [6, 7], by gently scrubbing the scalp sites by a piece of cotton and skin preparation gel and the electrodes with the electrode paste placed with the active electrode at the occipital scalp (Oz), the reference electrode at the frontal scalp (Fz), and grounded at the vertex (Cz). The electrode impedance was checked and kept ≤5kohm, and the impedance difference among electrodes was ≤3 kohm.

### 2.6. Statistical Analysis

The data were analyzed by using SPSS version 20. The one-way (ANOVA) test was used to test the significant differences between the three groups. Significant differences between each paired groups were then evaluated by the post hoc Tukey test to measure the lowest significant difference (LSD), and *P* value < 0.05 was considered statistically significant.

The proportions of normal and abnormal results were estimated by comparing with the control means of this study, i.e., the longest normal P100 latency was calculated by control mean + 2SE and the lowest normal P100 amplitude was calculated by control mean – 2SE.

## 3. Results

The baseline characteristics of the participants are presented in Table 1.

The test results of both eyes are presented together without right\left discrimination as there was no statistically significant difference in the mean values of the three parameters of both PRVEPs tests between the right and left eyes of each group. Additional tables show this in more details (Supplementary Tables 1 and 2).

Table 2 presents that, in the 60 min and 15 min tests, there was an increase in the mean values of P100 latency significantly and a decrease in the mean values of P100 amplitude significantly in group B as compared with those of group A and controls. The differences among group A and controls were also significant. With regard to N75 latency mean value, no statistical significant difference was detected among the three studied groups.

By calculating the upper limit of the normal P100 latency for the 60 min test (105.52) and for 15 min test (111.48) and the lower limit for normal P100 amplitude for the 60 min test (11.6) and for 15 min test (13.86), we can use them as the cutoff points between normal and abnormal results. The proportions of normal and abnormal P100 latency and amplitude of both tests are shown in Table 3.

As the ISCEV standards recommend, adult age ranges from 18 to 60 years and older than 60 years is considered as elderly age to be compared separately [6]. By dividing the three groups in two categories for each one according to the age as adult (40–60 yrs) and elderly (>60 yrs), we can evaluate them separately. In both 60 and 15 min PRVEPs, the difference was still statistically significant among controls and groups A and B in both age categories in relation to P100 latency and amplitude with the longest latency and lowest amplitude in group B. It is suggested that the differences between groups is not related to age difference.

Table 4 shows the results of the 60 and 15 min PRVEP test parameters in group A and group B according to good glycemic control (HbA1c < 7.5%) and poor glycemic control (HbA1c level ≥ 7.5%) [13].

Table 5 shows the results of the 60 and 15 min PRVEP test parameters in group A and group B according to the duration of T2DM.

## 4. Discussion

Although the main clinical diagnosis of DR is based on subjective detection of microvascular changes, the functional test as electrophysiological measures has the potential to be an early alternative determinant [14]. According to Hari Kumar et al. [15], the VEP changes were evident even in short-term hyperglycemia in gestational DM and T2DM pregnant females in comparison with normal glycemic pregnant females in spite of all being free from DR.

In this study, both 60 min and 15 min test results of group A revealed a statistically significant delay in the P100 latency and a decrease in the P100 amplitude when compared with the controls results, and these results were in accordance with those of Gupta et al. [16] for the 60 min test and with those of Heravian et al. [17] for the 15 min test. In addition, the presence of early NPDR clinical findings in group B was associated with a more deranged PRVEP test parameters; these results were in accordance with other studies' results [17, 18]. These data exhibited that neurological alterations occurred prior to the development of clinically significant DR and was more altered in the presence of DR. Despite the fact that Daniel et al. [19] who used mid-size checks (24–32 min) detected a significant delay in P100 latency, they did not find any significant decrease in P100 amplitude. This may be attributed to factors affecting the P100 amplitude as it is more influenced by technical factors and subject cooperation than the P100 latency [7].

In both tests results of group A and B, the proportions of abnormal P100 latency were higher than those of P100 amplitude with higher abnormal proportions in 15 min test. These proportions were greater than those measured in other studies [17, 18]. This variability could be explained by variation in the inclusion and exclusion criteria, DR diagnosis, recording conditions, and stimulus parameters.

As the proportions of abnormal P100 latency for group A (96%) and group B (100%) in the 15 min test were higher than those of the 60 min test, this could suggest that the foveal region is affected much earlier by DM and more altered by the presence of DR changes than the of the parafoveal region, unlike Balta et al. [20] who found a significant difference in P100 latency only in 60 min check size and no significant difference in other check sizes that he tested in the right eye of the diabetic patients with no DR.

Also, as the latency is more affected than the amplitude in group A, this mainly resembles multiple sclerosis features. And, in group B, the presence of early NPDR clinical features was associated with a more delay in the P100 latency and a more decrease in P100 amplitude in both tests, and these results also follow the VEP changes in multiple sclerosis, in which the VEPs are progressively delayed, and then as demyelination progresses, the amplitude will be attenuated [5]. Thus, early diabetic neural involvement seems to be a conductive damage at the myelin sheath level of optic nerve fibers [17]. These results contradict the ischemic optic neuropathy VEP findings as it mainly reduces the P100 amplitude with a much lower effect on the P100 latency than demyelination does [21].

The changes in the myelin sheath of the optic nerve is stated for the first time in experimental diabetes by Fernandez et al. [22], who identified extensive myelin irregularities and axonal loss with oligodendrocyte and astrocyte abnormalities at the distal portion of the optic nerve, and all were preceding retinal ganglion cell loss; these changes were detectable in animal models after only six weeks of diabetes. More recently, the reactive gliosis and neuronal apoptosis are hypothesized as n early DR processes, and these imply DR as a neurovascular complication [23]. These neural alterations were also detected anatomically by using spectral domain optical coherence topography (OCT) in many studies concluding a thinning in the inner retinal layer as a result of DM [24, 25]. Van Dijk et al. [26] reported that, in the eyes with minimal DR, there was a thinning in the retinal nerve fiber layer (RNFL), inner plexiform layer (IPL), and ganglion cell layer (GCL) of the pericentral zone of the macula, while in the peripheral zone of the macula, only the RNFL and IPL were thinner compared with normal eyes. These results are more suggestive that the foveal region is affected more than that of the parafoveal region and the loss of RNFL and IPL is preceding the loss of GCL.

Compared with other diabetic neuropathies, it seems to follow the same path as in polyneuropathy of peripheral nerves, as Valls-Canals et al. [27] concluded that the diabetic polyneuropathy is of two kinds: a demyelination which occurs with and without symptoms and an axonal loss which is the main cause of symptoms. DR pathology seems to be an actual central neuropathy similar to that of the peripheral nerves [3]. The perception of neural alterations as an early stage of DR proposes the possibility to find out other treatments to prevent vision loss [28]. In the nearest future, it is very likely that DR management will be established on neuroprotective agents [29].

In Tables 4 and 5, in both test results of group A, there are higher amplitudes detected in patients with good glycemic control and with less than ≤5 yrs DM duration, whereas the difference was nonsignificant in the P100 latency results. However, the latency is significantly prolonged in group B with poor glycemic control only on 15 min test. These results contrast Heravian et al.'s [17] results where they found no significant difference in the PRVEP parameters with the duration and glycemic status of DM. However, their study depended on FPG to assess the glycemic status of the patients, whereas the gold standard investigation to assess the glycemic status is by measuring the HbA1c level [30].

As the P100 latency showed no significant difference in group A according to the duration and glycemic status of T2DM in both PRVEP tests, this could indicate that the retinocortical conduction is affected early by DM and unrelated to glycemic status, whereas the P100 amplitude is affected by the increase in the DM duration and poor glycemic control. While in group B, the poor glycemic control was associated with more conduction delay in 15 min test, indicating a higher damaging effect of hyperglycemia on the retinocortical conduction affecting mostly the foveal region.

## 5. Conclusions

Collectively, the results of PRVEPs tests in this study are highly confirmative to the presence of neural alteration in the retina and/or optic nerve, before any clinically diagnosed DR changes, mainly as a conductive defect. In addition, these tests are noninvasive, quick, objective, cheap, and do not require mydriasis. Therefore, PRVEP tests could be considered as a valid tool to detect any early neurological changes which could be of great value in the prevention of permanent neuronal loss and blindness. In addition, the results of the 60 min test were not the same as the results of the 15 min test in both patients' groups; these could indicate that the T2DM effect on the different parts of the retina is not similar with more impact on the foveal region.

## 6. Recommendations

Further studies are required with the simultaneous use of pattern electroretinography (PERG) and PRVEP tests to distinguish between the purely optic nerve changes from those of the retinal abnormality origin, in addition to the use of OCT angiography to evaluate any subclinical macular edema.

## 7. Limitations

1. In the ophthalmology consultant in the Almawani Teaching Hospital, unfortunately, the PERG software needs an update setup in the VEP machine. Also, the OCT angiography is not available in the consultant.
2. Because of choosing to evaluate patients with T2DM and all are older than 40 years, this created a major difficulty to find subjects who are free from all the exclusion criteria. So, the number of candidates was limited to 50 in each group.

## Figures and Tables

**Table 1:** Baseline characteristics of the participants.

Variable		Controls *N* = 50	Group A *N* = 50	Group B *N* = 50	^*∗*^ *P* value
Age (mean ± SD)	40-60 yrs	50.5 ± 3.6	54.7 ± 4.2	55.7 ± 4.1	0.001
		*N* = 31 (62%)	*N* = 30 (60%)	*N* = 27 (54%)	—
	>60 yrs	63.5 ± 3.3	64.1 ± 3.1	65.7 ± 4.5	0.022
		*N* = 19 (38%)	*N* = 20 (40%)	*N* = 23 (46%)	—
Sex (male/female)		25/25	24/26	26/24	—
BP (mmHg) (mean ± SD)	Systolic	125.5 ± 11.3	122.8 ± 10.8	122 ± 10	0.08
	Diastolic	78.5 ± 8.1	77.2 ± 9.3	79.5 ± 9	0.182
BMI (Kg/m^2^) (mean ± SD)		30.7 ± 5.4	29.4 ± 5.1	28.7 ± 3.8	0.01
FPG (mg/dl)		88.6 ± 9.9	163.8 ± 30.8	177.6 ± 34	0.001
HbA1c (%)		4.07 ± 0.6	8.5 ± 1.7	9.4 ± 2.8	0.001
T2DM duration (mean ± SD)		—	7.7 ± 9.7	9.7 ± 4.4	0.001

BP: blood pressure, BMI: body mass index, FPG: fasting plasma glucose, HbA1c: glycated hemoglobin.

**Table 2:** The PRVEP tests results of both eyes in each group.

	Parameters	Control *N* = 100 eyes	Group A *N* = 100 eyes	Group B *N* = 76 eyes	LSD and *P* value
60 min PRVEP test	N75 Latency (ms)	68.3 ± 0.67^a^	69.4 ± 0.67^ab^	71 ± 0.96^b^	3.01
					*P* ^1^=0.276
					*P* ^2^=0.006
					*P* ^3^=0.084
	P100 Latency (ms)	104.32 ± 0.6^c^	108.63 ± 0.58^b^	117.5 ± 0.9^a^	4.31
					*P* ^1^=0.001
					*P* ^2^=0.001
					*P* ^3^=0.001
	P100 Amplitude (*μ*V)	12.6 ± 0.5^a^	10.4 ± 0.46^b^	8.2 ± 0.46^c^	2.14
					*P* ^1^=0.001
					*P* ^2^=0.001
					*P* ^3^=0.003

15 min PRVEP test	N75 Latency (ms)	82.57 ± 0.63	81.8 ± 1.1	79.6 ± 1.3	*P* ^1^=0.615
					*P* ^2^=0.053
					*P* ^3^=0.141
	P100 Latency (ms)	110.4 ± 0.54^c^	121.5 ± 0.58^b^	127.2 ± 0.45^a^	5.7
					*P* ^1^=0.001
					*P* ^2^=0.001
					*P* ^3^=0.001
	P100 Amplitude (*μ*V)	15.35 ± 0.73^a^	11 ± 0.54^b^	7.7 ± 0.55^c^	3.05
					*P* ^1^=0.001
					*P* ^2^=0.001
					*P* ^3^=0.001

Values are expressed as mean ± SE. Different letters represent significant difference at (*P* value < 0.05); LSD: lowest significant difference between the three groups. *P*^1^=*P*value between controls and group A; *P*^2^=*P*=*P* value between controls and group B; *P*^3^=*P* value between group A and group B.

**Table 3:** The proportion of normal and abnormal PRVEPs test results.

Parameters		Results	Controls	Group A	Group B
60 min PRVEP test	P100 latency	Normal	*N* = 58 (58%)	*N* = 24 (24%)	*N* = 5 (6.6%)
		Abnormal	*N* = 42 (42%)	*N* = 76 (76%)	*N* = 71 (93.4%)
	P100 amplitude	Normal	*N* = 51 (51%)	*N* = 39 (39%)	*N* = 15 (19.7%)
		Abnormal	*N* = 49 (49%)	*N* = 61 (61%)	*N* = 61 (80.3%)

15 min PRVEP test	P100 latency	Normal	*N* = 50 (50%)	*N* = 4 (4%)	*N* = 0 (0%)
		Abnormal	*N* = 50 (50%)	*N* = 96 (96%)	*N* = 76 (100%)
	P100 amplitude	Normal	*N* = 49 (49%)	*N* = 24 (24%)	*N* = 8 (10.5%)
		Abnormal	*N* = 51 (51%)	*N* = 76 (76%)	*N* = 68 (89.5%)

**Table 4:** The 60 min and 15 min PRVEP tests parameters in group A and group B according to good glycemic control and poor glycemic control (mean ± SD).

Groups according to the HbA1c level		60 min PRVEP test		15 min PRVEP test	
		P100	P100	P100	P100
		Latency (ms)	Amplitude (*μ*V)	Latency(ms)	Amplitude (*μ*V)
Group A	<7.5% (*N* = 28)	109.5 ± 5.6	12.8 ± 5	120.7 ± 5.5	13.8 ± 5.2
	≥7.5% (*N* = 72)	108.3 ± 6	9.4 ± 4.1	121.7 ± 6	9.8 ± 5
^*∗*^ *P* value		0.337	0.001	0.424	0.001
Group B	<7.5% (*N* = 12)	114.7 ± 7	6.8 ± 2.3	123.6 ± 6.3	8.4 ± 4.8
	≥7.5% (*N* = 64)	118 ± 8	8.5 ± 4.2	127.2 ± 3	7.5 ± 4.8
^*∗*^ *P* value		0.196	0.176	0.001	0.537

^*∗*^ *P* value < 0.05 considered statistically significant.

**Table 5:** The 60 min and 15 min PRVEP test parameters in group A and group B according to the duration of T2DM (mean ± SD).

Groups according to T2DM duration		60 min PRVEP test		15 min PRVEP test	
		P100	P100	P100	P100
		Latency (ms)	Amplitude (*μ*V)	Latency (ms)	Amplitude (*μ*V)
Group A	≤5 yrs *N* = 26	108.1 ± 3.8	13 ± 4.1 a	121.2 ± 5	14.3 ± 6.2 a
	6–10 yrs *N* = 38	108 ± 5.1	9.3 ± 4.1 b	121.3 ± 6.5	10 ± 4.4 b
	>10 yrs *N* = 36	109.7 ± 7.5	9.6 ± 5 b	121.9 ± 5.8	9.5 ± 4.8 b
^*∗*^ *P* value		0.383	0.004	0.877	0.001
Group B	≤5 yrs *N* = 16	119.5 ± 8.2	9.2 ± 3	126.7 ± 3.2	9.3 ± 4.4
	6–10 yrs *N* = 19	118.4 ± 8.3	8.1 ± 3.6	128.5 ± 2.7	7.1 ± 4.2
	>10 yrs *N* = 41	116.3 ± 7.8	8 ± 4.5	126.7 ± 4.5	7.3 ± 5.1
^*∗*^ *P* value		0.337	0.541	0.225	0.325

^*∗*^ *P* value < 0.05 considered statistically significant. Different letters represent significant difference at *P* value < 0.05).

## Data Availability

The datasets used and analyzed during the current study are available from the corresponding author upon request.

## Abbreviations

DR: Diabetic retinopathy
DM: Diabetes mellitus
VEPs: Visual evoked potentials
MRI: Magnetic resonance imaging
PRVEPs: Pattern-reversal VEPs
ISCEV: International Society for Clinical Electrophysiology of Vision
T2DM: Type2 diabetes mellitus
NPDR: Nonproliferative diabetic retinopathy
T1DM: Type1 diabetes mellitus
BP: Blood pressure
FPG: Fasting plasma glucose
HbA1c: Glycated hemoglobin
IOP: Intraocular pressure
BMI: Body mass index
LSD: Lowest significant difference
OCT: Optical coherence topography
RNFL: Retinal nerve fiber layer
IPL: Inner plexiform layer
GCL: Ganglion cell layer.

## Supplementary Materials

Supplementary Materials Supplementary Figure (1): fundoscopic pictures of (a) normal retina, (b) mild NPDR, (c) moderate NPDR, (d) sever NPDR with macular edema, and (e) advance PDR. Supplementary Table (1): the parameters of 60 min PRVEP test of right and left eyes of each group (mean ± SD). Supplementary Table (2): the parameters of 15 min PRVEP test of right and left eyes of each group (mean ± SD).
