# Supplementary material for: Pattern-Reversal Visual Evoked Potentials Tests in Persons with Type 2 Diabetes Mellitus with and without Diabetic Retinopathy
Source: Neurol Res Int. 2020 Aug 24;2020:1014857. doi: 10.1155/2020/1014857 (PMC7463399; doi:10.1155/2020/1014857)
Supplement: Supplementary Materials — Supplementary Figure (1): fundoscopic pictures of (a) normal retina, (b) mild NPDR, (c) moderate NPDR, (d) sever NPDR with macular edema, and (e) advance PDR. Supplementary Table (1): the parameters of 60 min PRVEP test of right and left eyes of each group (mean ± SD). Supplementary Table (2): the parameters of 15 min PRVEP test of right and left eyes of each group (mean ± SD). [file 1014857.f1.zip › 1014857.f1/Supplementary figure 1.docx]

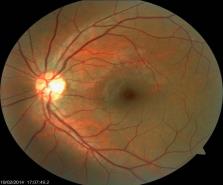

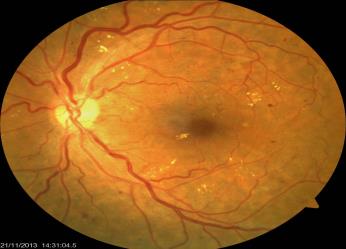

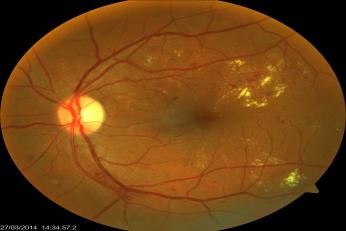

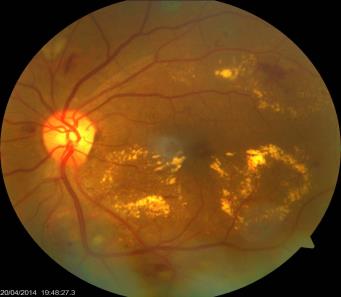

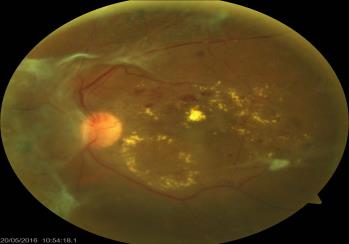


**(a)**

**(b)**

**(c)**

**(d)**

**(e)**

**Supplementary figure (1): fundoscopic pictures of (a) normal retina, (b) mild NPDR, (c) moderate NPDR, (d) sever NPDR with macular edema, and (e) advance PDR**

The clinical classification of DR which established by Wisconsin Epidemiologic Study of Diabetic Retinopathy (WESDR) whom construct the International Clinical Disease Severity Scale for DR and DME (Wilkinson et al., 2003). This classification represents a universal guideline to enable communication among ophthalmic caregivers, it is simple and easy to remember and founded on scientific trials, It does not need specific investigations such as optical coherence tomography (OCT) or fluorescein angiography (FAG).
