# Supplementary material for: Pattern-Reversal Visual Evoked Potentials Tests in Persons with Type 2 Diabetes Mellitus with and without Diabetic Retinopathy
Source: Neurol Res Int. 2020 Aug 24;2020:1014857. doi: 10.1155/2020/1014857 (PMC7463399; doi:10.1155/2020/1014857)
Supplement: Supplementary Materials — Supplementary Figure (1): fundoscopic pictures of (a) normal retina, (b) mild NPDR, (c) moderate NPDR, (d) sever NPDR with macular edema, and (e) advance PDR. Supplementary Table (1): the parameters of 60 min PRVEP test of right and left eyes of each group (mean ± SD). Supplementary Table (2): the parameters of 15 min PRVEP test of right and left eyes of each group (mean ± SD). [file 1014857.f1.zip › 1014857.f1/Supplementary Tables 1&2.docx]

**Supplementary Table (1): The parameters of 60min. PRVEPs test of right and left eyes of each group (mean ± SD)**

| 60min. PRVEPs test parameter | Controls  N=100eyes | | Group A  N=100eyes | | Group B  N=76 eyes | |
| --- | --- | --- | --- | --- | --- | --- |
|  | **Right N=50** | **Left**  **N=50** | **Right N=50** | **Left**  **N=50** | **Right N=37** | **Left**  **N=39** |
| N75  Latency (ms) | 68 ± 6.9 | 68.5 ±6.5 | 69.3 ±6.8 | 69.5 ±6.5 | 71.2 ± 9 | 71.3 ± 8 |
| *P-Value | 0.728 | | 0.915 | | 0.963 | |
| P100  Latency (ms) | 104.2±5.8 | 104.4±6.2 | 108.7±5.6 | 108.5±6.1 | 117± 8.6 | 118 ± 7.5 |
| *P-Value | 0.884 | | 0.870 | | 0.619 | |
| P100 Amplitude (μV) | 13.3 ± 5.3 | 11.8± 4.7 | 10.5± 4.4 | 10.3 ± 5 | 8.2 ± 4 | 8.3 ± 4.1 |
| *P-Value | 0.164 | | 0.731 | | 0.911 | |

*P-Value < 0.05 considered statistically significant.

**Supplementary Table (2): The parameters of 15min. PRVEPs test of right and left eyes of each group (mean ± SD)**

| 15min. PRVEPs test parameters | Control  N=100 eyes | | Group A  N=100 eyes | | Group B  N=76 eyes | |
| --- | --- | --- | --- | --- | --- | --- |
|  | **Right N=50** | **Left N=50** | **Right N=50** | **Left N=50** | **Right N=37** | **Left N=39** |
| N75  Latency(ms) | 82.4±6.03 | 82.7±6.7 | 81.2±11.2 | 82.5±10.8 | 78.6±11.9 | 80.6±11.4 |
| *P-Value | 0.820 | | 0.573 | | 0.468 | |
| P100 Latency(ms) | 110.4±5.3 | 110.4±5.6 | 121.5±6 | 121.4±5.7 | 126.5±4.02 | 127.8±3.8 |
| *P-Value | 0.974 | | 0.942 | | 0.143 | |
| P100 Amplitude (μV) | 15.7±7.3 | 15±7.5 | 11.01±5.3 | 10.8±5.5 | 7.5±5.1 | 7.8±4.5 |
| *P-Value | 0.616 | | 0.898 | | 0.830 | |

*P-Value < 0.05 considered statistically significant.

There was no statistically significant difference in the mean values of the three parameters of both PRVEPs tests between the right and left eyes of each group.
